# Development of an Immunoassay for Detection of Staphylococcal Enterotoxin-Like J, A Non-Characterized Toxin

**DOI:** 10.3390/toxins10110458

**Published:** 2018-11-06

**Authors:** Hisaya K. Ono, Nobuaki Hachiya, Yasunori Suzuki, Ikunori Naito, Shouhei Hirose, Krisana Asano, Katsuhiko Omoe, Akio Nakane, Dong-Liang Hu

**Affiliations:** 1 Department of Zoonoses, Kitasato University School of Veterinary Medicine, 35-1 Higashi-23-ban-cho, Towada, Aomori 034-8628, Japan; hisaono@vmas.kitasato-u.ac.jp (H.K.O.); wannsa_pana@yahoo.co.jp (N.H.); 2 Department of Microbiology and Immunology, Hirosaki University Graduate School of Medicine, 5 Zaifu-cho, Hirosaki 036-8562, Japan; s.hirose@hirosaki-u.ac.jp (S.H.); krisana@hirosaki-u.ac.jp (K.A.); a27k03n0@hirosaki-u.ac.jp (A.N.); 3 Department of Microbiology, Tokyo Metropolitan Institute of Public Health, Shinjuku-ku, Tokyo 169-0073, Japan; Yasunori_1_Suzuki@member.metro.tokyo.jp; 4 Department of Veterinary Medicine, Faculty of Agriculture, Iwate University, Ueda 3-18-8, Morioka, Iwate 020-8550, Japan; iku-rcn@i.softbank.jp (I.N.); s.h.foodsafety@gmail.com (K.O.)

**Keywords:** staphylococcal enterotoxin, immunoassay, ELISA, food poisoning, superantigenic activity

## Abstract

Staphylococcal enterotoxins (SEs) are the cause of staphylococcal food poisoning (SFP) outbreaks. Recently, many new types of SEs and SE-like toxins have been reported, but it has not been proved whether these new toxins cause food poisoning. To develop an immunoassay for detection of SE-like J (SElJ), a non-characterized toxin in SFP, a mutant SElJ with C-terminus deletion (SElJ∆C) was expressed and purified in an *E. coli* expression system. Anti-SElJ antibody was produced in rabbits immunized with the SElJ∆C. Western blotting and sandwich enzyme-linked immunosorbent assay (ELISA) detection systems were established and showed that the antibody specifically recognizes SElJ without cross reaction to other SEs tested. The limit of detection for the sandwich ELISA was 0.078 ng/mL, showing high sensitivity. SElJ production in *S. aureus* was detected by using the sandwich ELISA and showed that *selj*-horboring isolates produced a large amount of SElJ in the culture supernatants, especially in that of the strain isolated from a food poisoning outbreak in Japan. These results demonstrate that the immunoassay for detection of SElJ is specific and sensitive and is useful for determining the native SElJ production in *S. aureus* isolated from food poisoning cases.

## 1. Introduction

Staphylococcal enterotoxins (SEs), which are produced by *Staphylococcus aureus*, exhibit emetic activity in primates and are the causative agents of food poisoning cases in humans [1]. These toxins are also superantigens, which have the ability to stimulate a large population of T cells bearing specific Vβ elements [2]. Five major serological types of classical SEs, SEA to SEE, have been characterized [1], and many new types of SEs and SE-like (SEl) toxins (SEG to SElV, SElX, and SElY) have recently been reported [1,3,4,5,6,7]. Previous studies have demonstrated that staphylococcal food poisoning was almost induced by the classical SEs, such as SEA, SEB, and/or SEC [1]. In recent years, however, several *S. aureus* isolates derived from food poisoning do not possess classical SE genes but only have “new SE” genes, indicating that the recently described SEs and SEls could be also the causative agents of food poisoning and play important roles in the virulence of *S. aureus* [8,9,10].

*selj* was found as a gene located with several other SE genes, *selj* with *sed* and *ser*, or *selj* with *ser*, *ses*, and *set*, in the same pathogenic plasmids in *S. aureus* isolated from food poisoning cases [3,11]. The toxin proteins of SED, SER, SES, and SET have been characterized in their emetic and superantigenic activities and reported to be involved in staphylococcal food poisoning [1,4]. However, whether the *selj*-encoding protein SElJ is able to be produced and secreted in the *S. aureus* and whether the SElJ has emetic and/or superantigenic activities are still unclear. To investigate the biological characteristics of SElJ and its potential risk for food poisoning, we firstly prepared a recombinant SElJ and analyzed its biological properties and then developed an immunoassay, sandwich ELISA, for detection of SElJ and determined the SElJ production of *S. aureus* isolates from food poisoning outbreaks. The optimized sandwich ELISA showed high specificity and sensitivity for detection of SElJ and is successfully applied for determination of SElJ production in *S. aureus*. In addition, *selj*-harboring *S. aureus* produced a large amount of SElJ, indicating that SElJ could be an important risk factor involving in food poisoning outbreak.

## 2. Results

### 2.1. C-Terminus-Depleted SElJ Was Expressed and Purified

**Figure 1:**
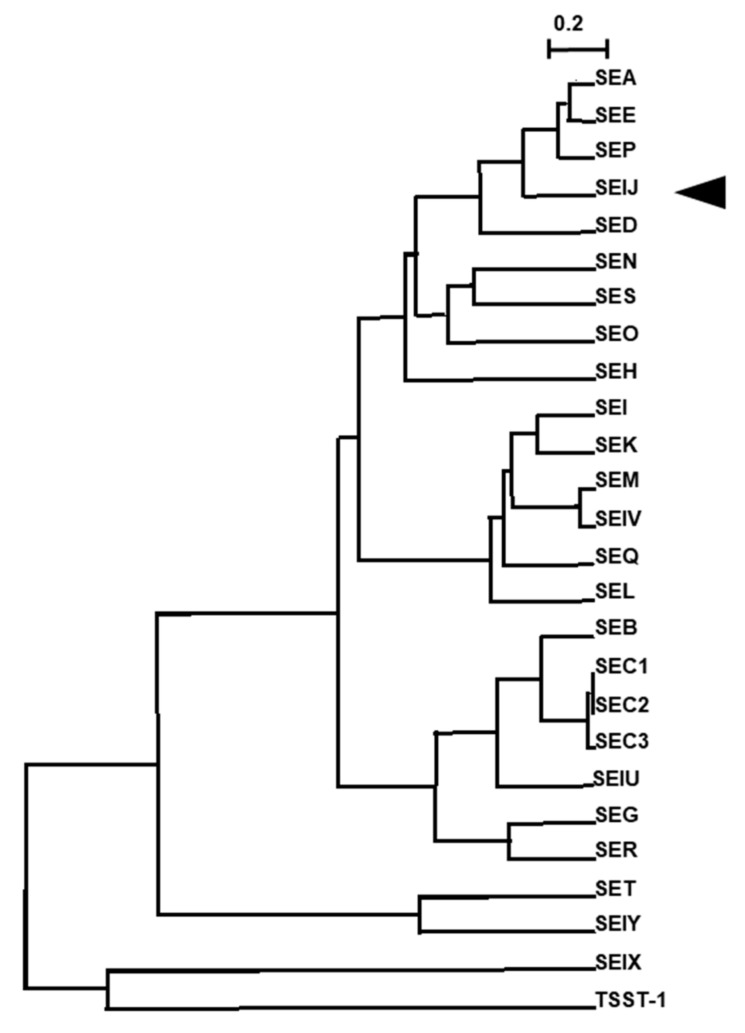
Phylogenic analysis of staphylococcal enterotoxins (SEs) and staphylococcal enterotoxin-like toxins (SEls). Multiple alignments and the construction of the phylogenetic tree of amino acid sequence of SEs and SEls were performed using ClustalW software. SElJ is closely related to SEA, SED, SEE, and SEP. Scale bar means a difference of 20% amino acid residues.

**Figure 2:**
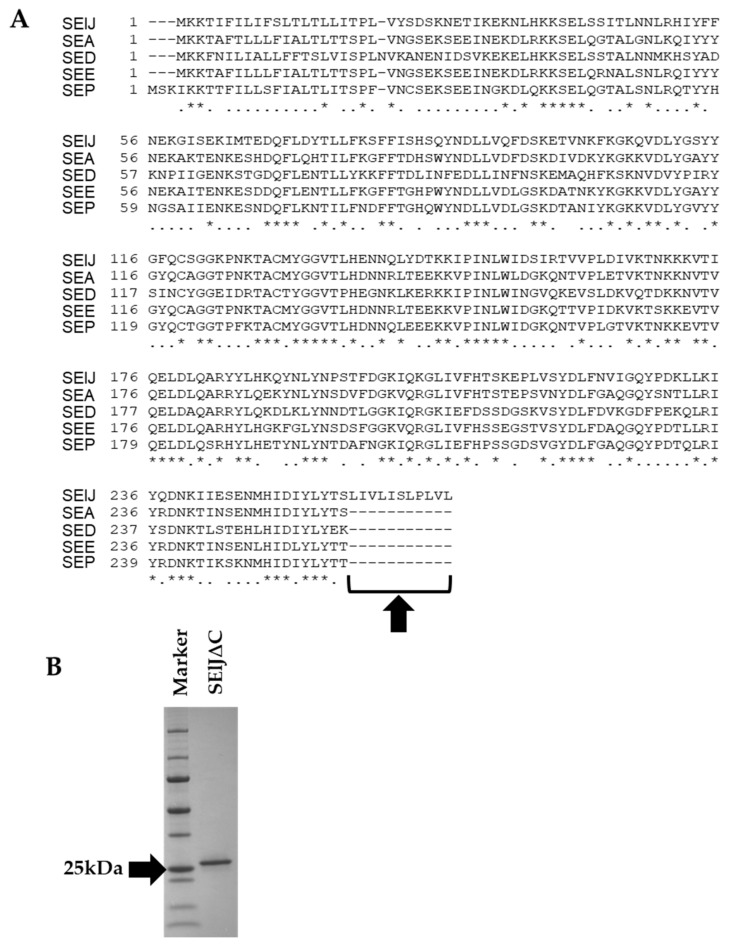
Deletion mutant of Staphylococcal enterotoxin-like J (SElJ). (**A**) SElJ has additional 11 hydrophobic amino acid residues at C-terminus compared with SEA, SED, SEE, and SEP. Arrow shows additional amino acids. Accession numbers of amino acid sequences are WP_000750881.1 (SElJ), AUU66069.1 (SEA), P20723.1 (SED), WP_000750405.1 (SEE), and WP_000034846.1 (SEP). (**B**) SDS-PAGE analysis of recombinant SElJ∆C. The purity was checked using Coomassie blue staining.

The amino acid sequence of SElJ is closely related to SEA, SED, SEE, and SEP and belongs to the same subgroup as these SEs in phylogenetic tree (Figure 1). However, SElJ has an additional hydrophobic region consisting of 11 amino acid residues in C-terminus as compared with SEA, SED, SEE, and SEP (Figure 2A). We tried several conditions to express soluble rSElJ and refold rSElJ from inclusion body, but we could not successfully prepare a soluble form of rSElJ. It is considered that the rSElJ expressed in *E. coli* cells aggregated and formed inclusion body due to the hydrophobic region in the C-terminus of the SElJ molecule. Therefore, we constructed SElJ expression vector excluding the C-terminal 11 amino acid residues. The deletion mutant recombinant SElJ was markedly expressed and prepared as a soluble protein with high purity, named as SElJ∆C (Figure 2B).

### 2.2. SElJ∆C Has Superantigenic Activity in Mouse Splenocytes

**Figure 3:**
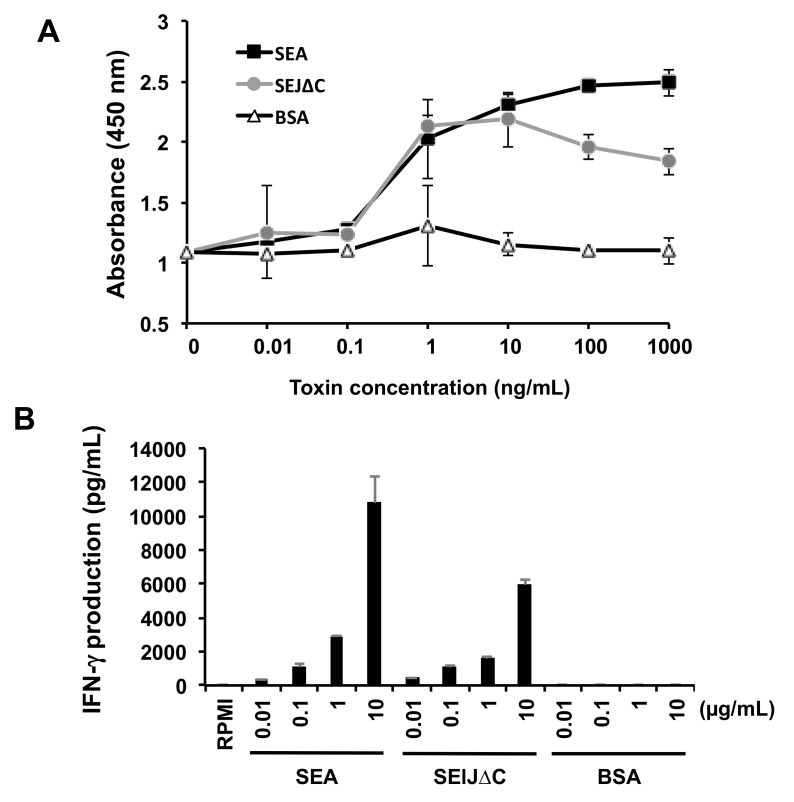
The superantigenic activity of SElJ with C-terminus deletion (SElJ∆C) in mouse splenocytes. Mouse splenocytes were incubated with various concentrations of SEA, SElJ∆C, and BSA for 48 or 72 h. (**A**) Measurement of cell proliferation was performed using Cell Counting kit-8. SElJ∆C induced mouse splenocyte proliferation, comparable with SEA. (**B**) IFN-γ production in culture media was determined by sandwich ELISA. Each bar represents the mean ± standard deviation of triplicate samples from a representative experiment. These data were reproducible in the three experiments.

To assess the superantigenic activity of SElJ∆C, proliferation of mouse splenocytes was measured. Mouse splenocytes were stimulated with SElJ∆C, using SEA or BSA as control proteins. The mitogenic activity of SElJ∆C was compared with SEA and BSA (Figure 3A). SElJ∆C induced proliferation of mouse splenocytes as well as SEA, and the minimum concentration of SElJ∆C and SEA to induce splenocyte proliferation was 1 ng/mL, whereas BSA exhibited no activity. To further examine the superantigenic activity, we quantified IFN-γ production in the cultures of splenocytes that were stimulated by SElJ∆C, SEA, or BSA. SElJ∆C induced IFN-γ production in a dose-dependent manner as well as SEA (Figure 3B). These results suggest that the purified SElJ∆C has superantigenic activity.

### 2.3. Development of a Sensitive and Specific Immunoassay for Detection of SElJ

**Figure 4:**
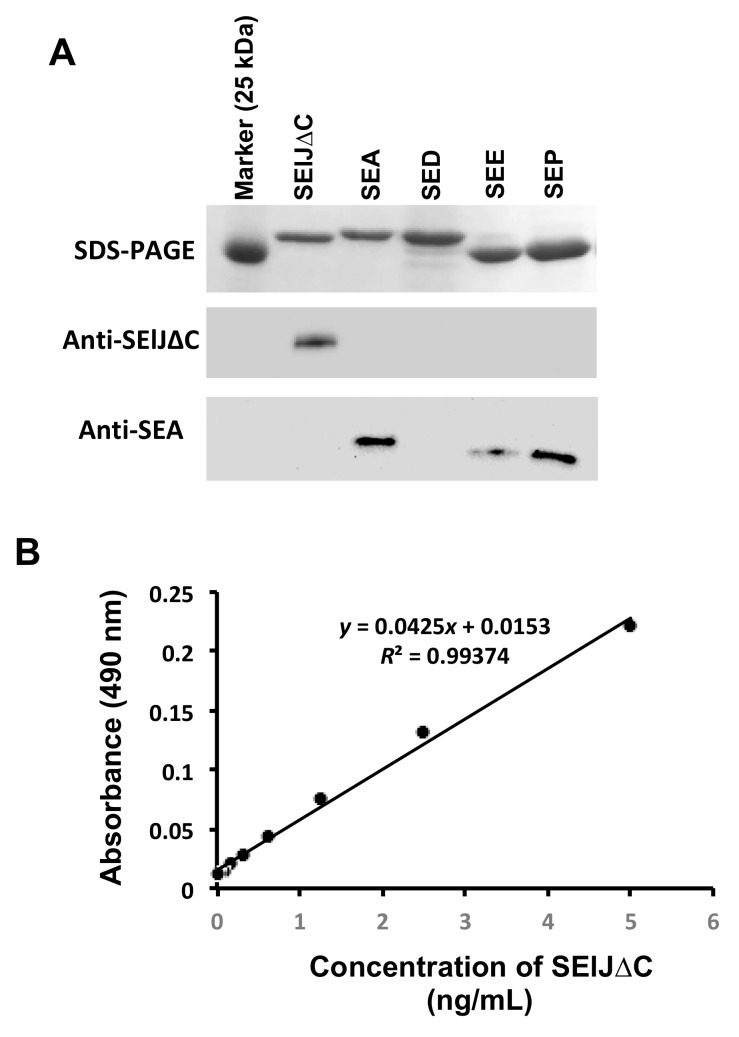
Specificity and sensitivity of anti-SElJ∆C antibody to SElJ∆C. (**A**) Western blotting using anti-SElJ∆C antibody. Anti-SElJ∆C antibody was confirmed its specificity to SElJ∆C, a recombinant protein, without cross reaction to SEA, SED, SEE, and SElP. (**B**) SElJ∆C was detected by sandwich ELISA. Linear standard curve with *R*^2^ between 0.99 and 1 was obtained for SElJ∆C when using SElJ∆C concentrations between 0.078 and 5 ng/mL.

Polyclonal antibody against SElJ∆C was prepared from the immunized rabbits and purified by affinity chromatography using SElJ∆C as an absorbing antigen. The specificity of the antibody was further analyzed by Western blotting using SEA, SED, SEE, and SEP as cross-reaction control toxins that closely resembled to SElJ. The results showed that purified antibody specifically recognizes SElJ∆C but shows no cross-reaction to other SEs tested (Figure 4A), indicating the anti-SElJ∆C antibody have high specificity to SElJ and is suitable to use for immunoassay.

To develop a high sensitive immunoassay that is capable of detecting SElJ in *S. aureus* culture supernatants, we established a SElJ detecting system by sandwich ELISA. The conditions of sandwich ELISA were optimized and the detection sensitivity and specificity were determined. For quantification purposes, SElJ∆C was used as standard. The detection limit of the immunoassay was 0.078 ng/mL and the linear range was 0.078–5 ng/mL (Figure 4B), showing high sensitivity.

### 2.4. Production of SElJ in S. aureus Strains Isolated from Food Poisoning

**Figure 5:**
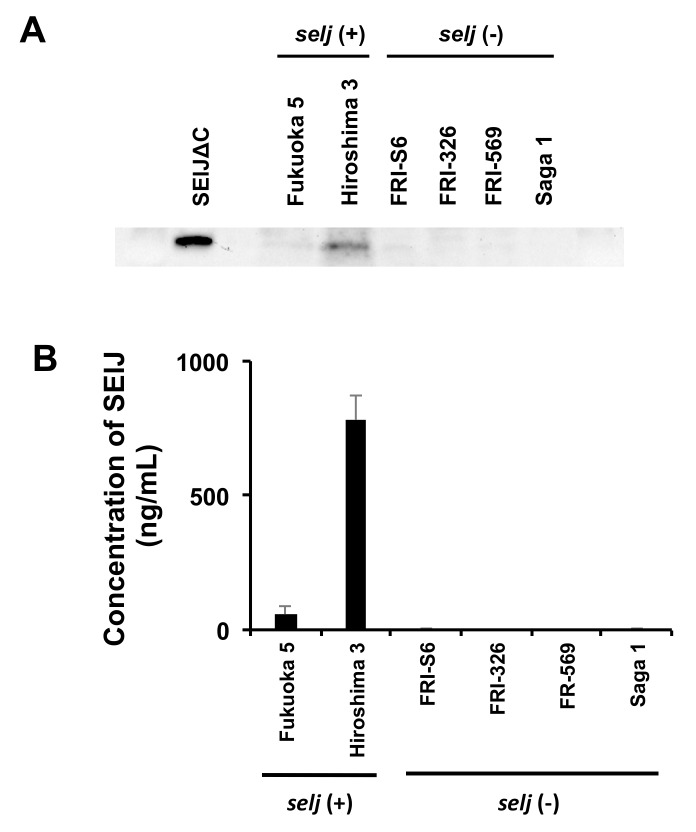
Detection of SElJ in *S. aureus* isolates derived from food poisoning. (**A**) Intact SElJ was detected in supernatant of *S. aureus* strains. (**B**) Evaluation of the SElJ productivity by sandwich ELISA. Hiroshima 3 strain, isolated from food poisoning outbreak, was produced a large amount of SElJ.

To analyze whether *selj*-harboring *S. aureus* produces SElJ toxin protein, the culture supernatant of *S. aureus* isolated from food poisoning outbreaks was detected by Western blotting with anti-SElJ∆C antibody. A protein band of the approximate size as that of SElJ∆C was detected in the supernatant of Hiroshima 3 strain, and a weak band was detected in the supernatant of Fukuoka 5 strain (Figure 5A).

To further quantify the production of *selj*-harboring *S. aureus*, we assessed the SElJ production in several *S. aureus* isolates using the sandwich ELISA. The results showed that Hiroshima 3 and Fukuoka 5, which are *selj*-positive food poisoning strains, produced significant SElJ at 778.1 ng/mL and 56.4 ng/mL in the culture supernatants, respectively (Figure 5B). In contrast, *selj*-negative strains, FRI-S6, FRI-326, FRI-569, and Saga 1, showed no production of SElJ in the supernatants. These results demonstrated that SElJ is expressed and secreted by *selj*-harboring *S. aureus* strains.

## 3. Discussion

SEs exhibit emetic activity in primates and cause food poisoning in humans. Recently, many new types of SEs (SEG-SEI, SEK-SET, etc.) and SE-like toxins (SElJ, SElU-SElY, etc.) have been reported. Investigations of foodborne outbreaks have also provided new insights into these newly identified toxins, but it has not been proved yet whether these new toxins cause food poisoning. Therefore, an increased understanding whether *S. aureus* isolates produce these newly identified toxins and cause food poisoning is urgent.

Although the *selj* gene was found in *S. aureus* strains isolated from food poisoning, it remained unclear whether SElJ protein can be produced by the bacteria and involved in staphylococcal food poisoning [8,12]. The lack of progress in studying the biological characteristics and emetic activity of SElJ can partially be attributed to the lack of convenient and appropriate detection methods, because SElJ could not be expressed and purified in the *E. coli* expression system. In the present study, we constructed an SElJ expression vector excluding the nucleic acid sequence, which encodes C-terminal 11 amino acid residues, a hydrophobic region in the C-terminus of SElJ molecule. The deletion mutant SElJ∆C was markedly expressed in *E. coli* cells and prepared as a soluble protein with high purity. This mutant showed strong immunogenicity that induced markedly specific antibody production in the immunized rabbits. The purified SElJ∆C also had superantigenic activity and immunoreactivity, indicating that the deletion mutant SElJ∆C still has biological and immunological activities. Using the purified SElJ∆C and rabbit antibody, we developed a sandwich ELISA system for detection of SElJ and showed that the detectable ranges between 0.078 and 5 ng/mL, demonstrating that the immunoassay had high sensitivity and specificity for SElJ.

Staphylococcal food poisoning is induced by some SEs produced by *S. aureus*, but not the bacteria themselves. Therefore, determination of SEs and/or SEls in foods and in the case of food poisoning outbreak is an indispensable method. Using our developed ELISA system to determine SElJ production in the culture supernatants of the bacteria isolated from food poisoning, the results showed that two strains, Hiroshima 3 and Fukuoka 5, exhibited significant toxin production in the culture supernatants. Especially, Hiroshima 3 was found to produce a large amount of SElJ at a concentration of 778.1 ng/mL. It is possible that SElJ played an important role in food poisoning outbreaks that were contributed by Fukuoka 5 and Hiroshima 3 strains. Previous studies demonstrated that the minimal food poisoning onset dose of classical SEs is 100 ng/person [13,14]. If the emetic activity of SElJ is comparable to that of classical SEs, the amount of SElJ could be sufficient for inducing food poisoning. To clarify the role of SElJ in staphylococcal food poisoning, it is necessary to further investigate the emetic activity of SElJ in emetic animal models, such as monkey and/or house musk shrew.

In conclusion, a mutant SElJ with C-terminus deletion (SElJ∆C), which is valuable for producing a specific antibody production, was constructed and purified. Using the antibody and SElJ∆C protein, we developed, for the first time, a sensitive and specific immunoassay for detection of native SElJ. Furthermore, it was proved that *S. aureus* strains isolated from food poisoning produce a large amount of SElJ, indicating that SElJ could be important risk factor and would be involved in food poisoning.

## 4. Materials and Methods

### 4.1. Bacterial Strains and Culture Conditions

A total of 6 *S. aureus* strains were used in this study (Table 1). Two strains (Fukuoka 5, Hiroshima 3), which harbor *selj*, were isolated from sample collected during food poisoning outbreaks in Japan. Four strains (FRI-S6, FRI-326, FRI-569 and Saga1) were reference strains without *selj*. *S. aureus* cultures for total DNA isolation were grown in Trypticase soy broth (Nissui, Tokyo, Japan) at 37 °C for 16 h with aeration. *S. aureus* cultures for SElJ production were grown in brain heart infusion broth (Difco Laboratories, Detroit, MI) supplemented with 1% yeast extract at 37 °C for 48 h with aeration [15].

### 4.2. Cloning and Preparation of SElJ

Multiple alignments and the construction of the phylogenetic tree of amino acid sequence of SEs and SEls were performed using ClustalW software (version 2.1, http://clustalw.ddbj.nig.ac.jp/) [17]. To construct the SElJ expression plasmids, PCR primers were designed to amplify the gene fragment corresponding to the mature form of the SElJ protein with or without 11 amino acid residues at C-terminus (forward; CCCCGGATCCAGCAAAAATGAAACAATTAAAG, reverse for full length; CCCCGAATTCCTACAGAACCAAAGGTAGAC, reverse for C-terminus deletion; CCCCGAATTCTTAGCTTGTATATAAATATATATC). Total DNA of the *S. aureus* Fukuoka 5 strain that harbors *selj*, *ser*, *ses*, and *set* genes was purified with QIAamp DNA Mini kit (QIAGEN, Tokyo, Japan) [4]. The *selj* gene was amplified and digested with *Bam*HI and *Eco*RI. The *selj* gene fragments were then cloned into pGEX6P-1, a glutathione S-transferase (GST) fusion vector. The expression and purification of the recombinant GST fusion proteins and the cleavage and removal of the GST tag from the SE proteins were performed by the methods described previously [4]. The resulting recombinant SElJ and SElJ with C-terminus deletion (SElJ∆C) had five additional amino acid residues, GPLGS, at the N terminus. The protein concentration was determined by the Bradford assay (Bio-Rad Laboratories, Hercules, CA, USA), using bovine serum albumin (BSA; Bio-Rad Laboratories) as a standard. Each of the recombinant protein bands was detected on sodium dodecyl sulfate-polyacrylamide gel electrophoresis (SDS-PAGE). The preparation of recombinant SEA, SED, SEE, and SEP have been described by our research group elsewhere [9,18].

### 4.3. Analysis of Superantigenic Activity of SElJ∆C

To determine the superantigenic activity of SElJ∆C, the mitogen activity and interferon-gamma (IFN-γ) production in mouse splenocytes stimulated by SElJ∆C were measured. Briefly, mouse spleens were isolated from C57BL/6J mice (female, 6-week-old), and the splenocytes were incubated at 37 °C for 48 h in 96-well round-bottomed tissue culture plates (Greiner Bio-One International, Kremsmünster, Austria) with different concentrations of SElJ∆C, SEA, or BSA, and then incubated with 10 µL/well of cell counting kit-8 solution (Dojindo Laboratories, Kumamoto, Japan) for 12 h. Absorbance at 490 nm of each well was measured. Data are presented as means ± standard deviation of triplicate determinations. On the other hand, splenocytes were incubated at 37 °C for 72 h with different concentrations of SElJ∆C, SEA, or BSA. The supernatant from the cultures was harvested for IFN-γ assay (R&D systems, Minneapolis, MN, USA) [19]. Data are presented as the mean ± standard deviation of triplicate experiments. The experiments were conducted in accordance with the Animal Research Ethics Committee, Kitasato University School of Veterinary Medicine, and followed the Guidelines for Animal Experimentation, Kitasato University.

### 4.4. Preparation of Anti-SElJ Antibody

Rabbit anti-SElJ∆C sera were prepared by SElJ∆C-immunized rabbits as previously reported method with minor modifications [20]. Titers of antibody were monitored by means of ELISA. The specific antibody was purified from the immune sera using HiTrap NHS-activated HP (GE Healthcare Japan, Tokyo, Japan), which was bound with SElJ∆C, according to the instructions. Animal experiments were conducted in accordance with the Animal Research Ethics Committee, Iwate University, and followed the Guidelines for Animal Experimentation, Iwate University.

### 4.5. Specificity of Anti-SElJ Antibody by Western Blot Analysis

To evaluate the specificity of the anti-SElJ antibody, the proteins of SElJ∆C, SEA, SED, SEE, SEP, and BSA were separated by SDS-PAGE and then transferred to nitrocellulose membranes (Bio-Rad Laboratories, Richmond, CA, USA) by the method as previously described [4,16]. Reactive signals were detected using a horseradish peroxidase-labeled protein A (Bio-Rad Laboratories) and Clarity western ECL substrate (Bio-Rad Laboratories) in accordance with the manufacturers’ instructions.

### 4.6. Development of Sandwich ELISA

ELISA was performed in 96-well microplates (MICROLON 96 well microplate, Greiner Bio-One). Each well was coated with 100 µL of anti-SElJ∆C antibody (2 µg/mL) in 0.05 M carbonate-bicarbonate buffer (pH 9.6, Sigma-Aldrich Japan, Tokyo, Japan) at 4 °C overnight. Then, each well was blocked with 250 µL/well of PBS/0.1% BSA, and the plate was immediately emptied. Afterward, 100 µL/well of samples or standards were added and incubated at 37 °C for 2 h. After washing, 100 µL/well of horse radish peroxidase labeled anti-SElJ∆C antibody (2 µg/mL), which was diluted in Can Get Signal Immunoreaction Enhancer solution 1 (TOYOBO, Osaka, Japan), was added and incubated at 37 °C for 1 h. After wash each well, 100 µL/well of 0.8 mg/mL *o*-phenylendiamine (Sigma-Aldrich, St. Louis, MO, USA) in 0.05 M phosphate-citrate buffer (Sigma-Aldrich) was added and incubated for 20 min, 100 µL/well of 2 N H_2_SO_4_ was added. The absorbance at 490 nm was read with a microplate reader (Model 680, Bio-Rad Laboratories). Toxin concentrations were determined by converting absorbance to the corresponding concentrations by use of the standard curve.

### 4.7. Detection of SElJ Production in S. aureus Isolates

To analyze and quantify SElJ production in *S. aureus* isolates, the culture supernatants from six *S. aureus* isolates were harvested and were then pre-incubated with Normal Rabbit Serum-binding PVDF membrane (GE Healthcare Japan) at 4 °C overnight to avoid any nonspecific reaction caused by Protein A. The samples were then diluted 10- or 100-fold in PBS/0.1% BSA. ELISA was performed in 96-well microplates (MICROLON 96 well microplate) as described above. Toxin concentrations in the culture supernatants were determined by converting absorbance to the corresponding concentrations of the standard curve.

## Figures and Tables

**Table 1:** *S. aureus* strains used in the present study.

*S. aureus* Strains	*se* Genotype	References
Fukuoka 5	*selj*, *ser*, *ses*, *set*	[3]
Hiroshima 3	*seg*, *sei*, *sem*, *sen*, *seo*, *selj*, *ser*, *ses*	this study
S6	*sea*, *seb*, *selk*, *seq*	[16]
FRI-326	*sea*, *seb*, *selk*, *seq*	[16]
FRI-569	*sea*, *seb*, *selk*, *seq*	[16]
Saga 1	*seg*, *sei*, *sem*, *sen*, *seo*, *sep*	[16]
